# An *N*-Acetyl Cysteine Ruthenium Tricarbonyl Conjugate Enables Simultaneous Release of CO and Ablation of Reactive Oxygen Species

**DOI:** 10.1002/chem.201502474

**Published:** 2015-08-28

**Authors:** João D Seixas, Miguel Chaves-Ferreira, Diana Montes-Grajales, Ana M Gonçalves, Ana R Marques, Lígia M Saraiva, Jesus Olivero-Verbel, Carlos C Romão, Gonçalo J L Bernardes

**Affiliations:** [a] Instituto de Medicina Molecular, Faculdade de Medicina da Universidade de Lisboa Av. Prof. Egas Moniz, 1649-028 Lisboa (Portugal) http://www.gbernardes-lab.com E-mail: gbernardes@medicina.ulisboa.pt; [b] Instituto de Tecnologia Química e Biológica-António Xavier, Universidade Nova de Lisboa Av da República, 2780-157 Oeiras (Portugal); [c] Alfama Ltd., Instituto de Biologia Experimental e Tecnológica, IBET, Av. da República EAN, 2780-157 Oeiras (Portugal); [d] Department of Chemistry, University of Cambridge Lensfield Road, CB2 1EW Cambridge (UK) E-mail : gb453@cam.ac.uk; [e] School of Pharmaceutical Sciences, University of Cartagena Campus of Zaragocilla, Cartagena, Bolivar 130015 (Colombia)

**Keywords:** anti-oxidants, carbon monoxide, *N*-acetyl cysteine, prodrugs, reactive oxygen species, ruthenium

## Abstract

We have designed and synthesised a [Ru(CO)_3_Cl_2_(NAC)] pro-drug that features an *N*-acetyl cysteine (NAC) ligand. This NAC carbon monoxide releasing molecule (CORM) conjugate is able to simultaneously release biologically active CO and to ablate the concurrent formation of reactive oxygen species (ROS). Complexes of the general formulae [Ru(CO)_3_(L)_3_]^2+^, including [Ru(CO)_3_Cl(glycinate)] (CORM-3), have been shown to produce ROS through a water–gas shift reaction, which contributes significantly, for example, to their antibacterial activity. In contrast, NAC-CORM conjugates do not produce ROS or possess antibacterial activity. In addition, we demonstrate the synergistic effect of CO and NAC both for the inhibition of nitric oxide (formation) and in the expression of tumour-necrosis factor (TNF)-α. This work highlights the advantages of combining a CO-releasing scaffold with the anti-oxidant and anti-inflammatory drug NAC in a unique pro-drug.

Carbon monoxide releasing molecules (CORMs) have been demonstrated to be able to reproduce the biological effects of exogenously administered carbon monoxide (CO). Their ability to deliver therapeutically useful amounts of CO is now well established, and several in vitro and in vivo studies suggest their potential use as therapeutics.[1, 2] Most available CORMs are metal–carbonyl complexes, including enzyme-[3, 4] and light-triggered complexes,[5] vitamin B12 Re-based complexes,[6] or liver-targeted [Mo(CO)_3_(CNR)_3_] and [Ru(CO)_3_Cl_2_(thiogalactopyranoside)] complexes.[7, 8] The two most studied CORMs derive from a {Ru^II^(CO)_3_} scaffold: the DMSO-soluble [{Ru(CO)_3_Cl_2_}_2_] (CORM-2) and its water-soluble derivative *fac*-[Ru(CO)_3_Cl(κ^2^-H_2_NCH_2_CO_2_)] (CORM-3).[9] Once characterised as fast CO releasers, it is has now been shown that these CORMs are unable to transfer CO to deoxymyoglobin (deoxy-Mb) as previously accepted.[10] This correlates well with the absence of CO in the headspace of solutions of CORM-3 and other [Ru(CO)_3_Cl_2_(L)] (L=ligand) complexes, as determined by gas-chromatography (GC) methods.[11, 12] Yet, these two CORMs have attracted great interest due to their considerable biological effects in animal models of disease without increasing carboxyhemoglobin (CO-Hb) levels in circulation. For instance, CORM-3 has been shown to protect against myocardial infarction and heart failure[13, 14] as well as to help conservation of tissues for transplantation,[15] while CORM-2 was able to protect allogeneic aortic transplants in mice.[16]

Recent studies have demonstrated the importance of the nature of the ancillary ligand(s) in complexes of the general formulae [Ru(CO)_3_(L)_3_]^2+^ for their stability in aqueous media and subsequently on their CO release profile, cytotoxicity and anti-inflammatory properties.[17, 18] In addition, the reactivity of CORM-3 and [Ru(CO)_3_Cl_2_(thiazole)] was characterised in the presence of proteins, such as lysozyme, bovine serum albumin (BSA) or human transferrin.[11, 12, 18, 19] It has been observed that the products resulting from the hydrolytic decomposition of CORM-3 and [Ru(CO)_3_Cl_2_(thiazole)] react rapidly with histidine residues on proteins to generate protein–Ru adducts bearing two or one CO ligands, respectively. Furthermore, it has been recently shown that protein–[Ru^II^(CO)_2_] metalloproteins are capable of spontaneously releasing CO in aqueous solution, cells and animals.[20, 21] Finally, during the hydrolytic decomposition process, many of these CORMs generate reactive oxygen species (ROS) through a water–gas shift reaction (WGSR; Figure 1 A) that can also contribute to their biological activity, as is the case for CORM-3 bactericidal killing activity.[22]

**Figure 1:**
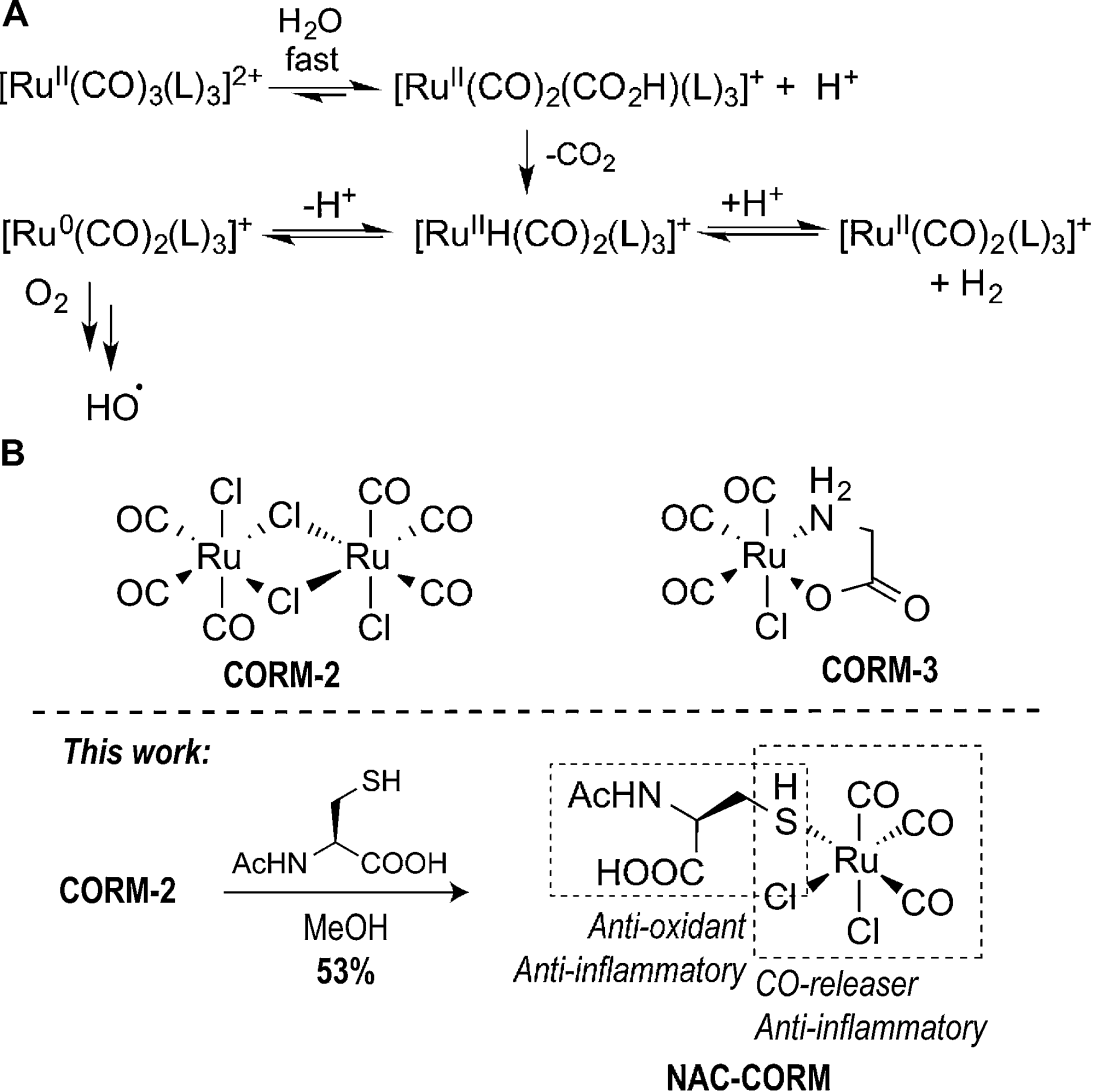
A) The hydrolytic instability of complexes of the general formulae [Ru^II^L_3_(CO)_3_]^2+^ that results in CO release but also formation of ROS can be explained by a water–gas shift mechanism. B) Structures of commonly used CORM-2 and CORM-3; reaction of CORM-2 with *N*-acetyl cysteine (NAC) in anhydrous methanol yields the complex NAC-CORM that features a CO releasing moiety and an anti-oxidant ligand.

Herein, we sought to use the knowledge derived from extensive studies of the stability, CO release and biological activity of many CORMs of general formulae [Ru(CO)_3_(L)_3_]^2+^ to design a CORM conjugate bearing a ligand that would deliver biologically active CO and scavenge the ROS known to be formed during the CO release process form [Ru(CO)_3_(L)_3_]^2+^ complexes. The ligand we chose to introduce into such a CORM conjugate was the drug *N*-acetyl cysteine (NAC) (Figure 1 B), a potent anti-oxidant and scavenger of hydroxyl radicals that has excellent anti-inflammatory activity.[23] We envisioned that the simultaneous release of CO and NAC could result in an enhanced anti-inflammatory activity, while NAC could also abolish any ROS formed during CO release. An identical strategy by combining two drugs with complementary activities, that is, the conjugation of cisplatin and aspirin, resulted in a synergistic effect towards the killing of cancer cells.[24, 25]

We started by synthesising the NAC-CORM conjugate complex through reaction of the commercially available CORM-2 dimer with NAC (Figure 1 B). The reaction occurs in a coordinating solvent (MeOH) that generates the solvated species [Ru(CO)_3_Cl_2_(HOMe)] prior to NAC substitution.[26] Analysis of the off-white powder isolated gives a stoichiometry that matches the adduct [Ru(CO)_3_Cl_2_(NAC)], which can also be a dimer or higher oligomer (see Supporting Information). The FTIR spectrum presents the usual *ν*_CO_ stretching band pattern corresponding to the *fac*-M(CO)_3_ fragment (see Supporting Information): a sharp, strong vibration at 2126 cm^−1^ and a very strong, broader band at 2062 cm^−1^. This indicates that the *fac*-Ru(CO)_3_ arrangement remained intact in the product and no nucleophilic addition to a coordinated CO has taken place.[18] The C=O signal from the amide is observed at 1749 cm^−1^. We suggest that the NAC ligand binds the {Ru(CO)_3_Cl_2_} moiety through the SH group, as documented in other [Ru^II^(L)_*n*_(SHR)] and [Ru^II^(L)_*n*_(SH_2_)] complexes.[27, 28] However, the *ν*_SH_ stretching vibration in the region around 2500 cm^−1^ is a broad peak probably reflecting hydrogen-bond type interactions in a non-monomeric structure of higher complexity. The ^1^H NMR spectrum of the NAC-CORM complex was acquired in CD_3_OD and D_2_O (see Supporting Information). The spectra in both solvents are very similar revealing a surprisingly good stability in aqueous solution. No SH or NH signals are observed. However, the spectra of pure NAC in the same solvents do not show the SH proton and the NH proton has only a very weak signal (see Supporting Information). The absence of the signals of the CH and CH_2_ protons of free NAC in both NAC-CORM spectra indicates that there is no ligand dissociation or contamination with excess of unreacted NAC. Integration of the two close CH_3_ signals in the acetyl region and the two close signals in the CH_2_ region gives a ratio of 3:2 protons, suggesting the presence of either isomers or a complex oligomeric structure with magnetically non-equivalent NAC ligands. The CH_2_ protons are deshielded relative to free NAC, whereas the CH protons are shielded, but are strongly split and could not be clearly assigned (see Supporting Information). Regardless of structural details, these data are in good agreement with FTIR and analytical data that confirm that the complex NAC-CORM contains the intact *fac*-Ru^II^(CO)_3_ fragment coordinated to the NAC ligand, thus carrying both the CO delivery and anti-oxidant functions.

We began by determining the rate of CO release of the aqueous soluble NAC-CORM conjugate to the headspace of a phosphate-buffered saline (PBS) pH 7.4 solution at room temperature and in the dark, using gas chromatography (GC) with a thermal conductivity detector (TCD, see the Supporting Information). Similar to analogous compounds of the formulae [Ru(CO)_3_(L)_3_]^2+^, under these conditions CO could not be detected in the headspace of the solution (Table 1). Instead, CO_2_ was slowly produced as the result of the extremely facile attack of HO^−^ at coordinated CO, followed by the water–gas shift reaction shown in the second step of the scheme in Figure 1 A.[11, 29] Also, similar to CORM-3, NAC-CORM did not raise the percentage of CO-Hb, when incubated in sheep blood at 37 °C, as measured by oximetric quantification (data not shown). Finally, we evaluated the cytotoxicity of NAC-CORM in RAW264.7 cells by using the MTT assay. It was found that NAC-CORM is not toxic up to a concentration of 100 μm (Table 1).

**Table 1:** Comparison of some physicochemical and biological properties of CORM-3 and NAC-CORM

CORM	*ν*_CO_ (KBr [cm^−1^])	Equiv CO^[b]^	Equiv CO_2_^[b]^	Cytotoxicity [μm]^[c]^
CORM-3^[a]^	2139 (s)	0	0.68	>100
	2057 (s)			
	1981 (w)			
NAC-CORM	2126 (s)	0	0.65	>100
	2062 (s)			

[a] CORM-3 was synthesised as previously described.[30] [b] Equivalents of CO and CO_2_ released in the headspace of a closed vial after incubation of CORMs in H_2_O after 24 h at room temperature under nitrogen and in the dark, as determined by GC-TCD. [c] Cytotoxicity of CORMs was tested in RAW264.7 cells (MTT assay; 24 h incubation; IC_50_).

In 2012, Chang and co-workers introduced the coat protein COP-1 as a CO specific organometallic probe that turns fluorescence on upon a selective reaction with CO through a palladium-mediated carbonylation reaction.[31] Importantly, they also showed that the fluorescence of COP-1 in buffer is turned on either by CO gas or by CORM-3. Again similarly to the case of CORM-3 we observed that in PBS (pH 7.4) the NAC-CORM complex triggered a robust fluorescence turn-on response; a tenfold increase within 120 min in comparison with the control—a solution of COP-1 (Figure 2 A). In the absence of CO, COP-1 is only weakly fluorescent.[25] When compared with CORM-3, NAC-CORM showed a slower CO release kinetics as detected by CO reaction with COP-1, in particular during the first 10 min of incubation with the fluorescent CO-selective probe (Figure 2 A). However, after 60 and 120 min no significant differences were detected with a similar maximum fluorescence observed for both CORMs.

**Figure 2:**
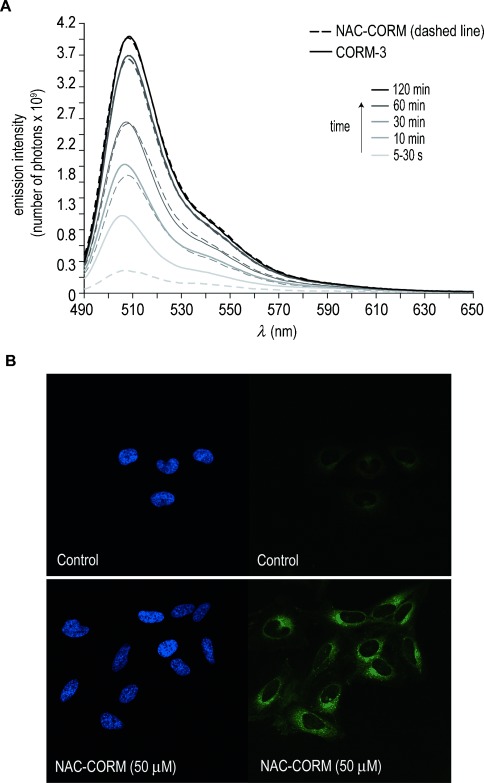
A) Comparison of CO release from NAC-CORM and CORM-3: CO release measurement using COP-1, read from 490 to 650 nm, following excitation (*λ*_ex_=475 nm). Photoemission spectra were taken at 5 to 30 s, 10, 30, 60 and 120 min after the addition of 1 μm COP-1 to 50 μm of NAC-CORM and CORM-3, respectively, in PBS pH 7.4 at 37 °C. B) Confocal microscopy images for cellular CO release in untreated (control) and treated HeLa cells (50 μm NAC-CORM). After an initial 30 min treatment with NAC-CORM, 1 μm COP-1 was added and following 30 min incubation period pictures were taken. In each panel, the left picture shows nuclear staining using Hoechst 33342 (blue) and the picture to the right shows COP-1 turn-on response to CO (green).

Next we used confocal microscopy to visualise changes in CO levels in HeLa cells after incubation with NAC-CORM. HeLa cells were incubated in the absence (control) or presence of 50 μm NAC-CORM, and then treated with COP-1 and a significant increase in intracellular fluorescence for cells incubated with NAC-CORM over the control was observed (Figure 2 B and Figure 1 in Supporting Information). In order to turn fluorescence on CO must be transferred from the coordination sphere of Ru to that of Pd. This can happen if the Ru complexes decompose and the CO liberated to the medium is captured by COP-1, or if COP-1 reacts directly with some Ru–CO species exchanging CO. Previous evidence points to the preferred decomposition of {Ru^II^(CO)_3_}-containing complexes according to the scheme in Figure 1 A. Proteins make adducts with [Ru(CO)_2_(H_2_O)_3_]^2+^ species, some of which are active CO delivery species.[11, 12, 18, 20, 21] However, it is not difficult to admit that the very labile coordination sphere of [Ru(CO)_2_(H_2_O)_*n*_]^2+^ type species may facilitate reaction with COP-1 and CO transfer. Fluorescence will be turned on and increase as long as more [Ru(CO)_2_(H_2_O)_*n*_]^2+^ species are formed. This process will consume all COP-1, which is the limiting reagent, while the decomposition of the CORM will proceed independently. Indeed, the longer it takes to achieve decomposition and formation of the [Ru(CO)_2_(H_2_O)_*n*_]^2+^ type species, the longer it will take for fluorescence to appear. The fact that NAC-CORM is actually slower than CORM-3 to generate fluorescence at early incubation times is not unexpected, since the stability of [Ru^II^(CO)_2_(CO_2_H)Cl_2_(L)]^−^ present in the first equilibrium of Figure 1 A, depends on the nature of L.[18] In this case NAC-CORM is more stable in aqueous solution than CORM-3, according to the ^1^H NMR data. If this process takes place intracellularly, the fluorescence reveals that the CORM has been taken-up by the cell and has started to liberate CO. Part of this CO will be scavenged by COP-1 and the rest will eventually trigger the desired biological effects.

Our data shows that the new water-soluble NAC-CORM conjugate is able to generate levels of CO in solution that are comparable to those produced with CORM-3, although with slower kinetics. The slower kinetics are likely driven from an increased stability provided by the Ru–S bond present in NAC-CORM that makes the hydrolysis and subsequent CO release slower. This is of particular importance for in vivo CO delivery applications, for which a controlled CO release profile is required. Incidentally, S-bound adducts [Ru(CO)_3_Cl_2_(S–R)] were shown to be among the most stable species to hydrolysis and CO loss in a series of [Ru(CO)_3_Cl_2_(L)] adducts with different C, N, O, and P donors.[18]

In this study we set ourselves to develop a [Ru(CO)_3_(L)_3_]^2+^ complex that would release CO and at the same time carry a ligand that would ablate ROS produced during CO release, while enhancing the anti-inflammatory properties of CO. After providing clear data for CO release both in solution and cells, we examined the levels of endogenously formed ROS in *E. coli* cells treated with either NAC-CORM or CORM-3 for 2 h (Figure 3 A). The fluorescence intensities (FI) are represented as the subtraction of untreated cultures from cultures exposed to either NAC-CORM or CORM-3 normalised in relation to the OD600 nm of the respective culture. The data reveals a significant increase of ROS content in cells exposed to CORM-3, but not in those exposed to NAC-CORM. After 2 h, cells treated with 100 μm of NAC-CORM displayed only 25 % of the ROS levels induced by CORM-3. This is in accordance with previous data showing that the ROS generated from the hydrolytic instability of CORM-2 could be abolished to similar levels of untreated cells by co-incubation with the ROS scavenger glutathione.[22] The generation of ROS has been shown to also contribute to the observed potent bactericidal activity of CORM-3.[22] Thus, we decided to perform a direct comparison of the antibacterial activity of NAC-CORM and CORM-3 to assess the effect of the presence of the anti-oxidant NAC ligand in bacterial survival (Figure 3 B). We observed that unlike CORM-3, treatment with NAC-CORM did not produce any significant effect on bacterial survival (Figure 3 B). Our data suggests that the presence of the ROS scavenger NAC ligand in the conjugate is able to ablate the ROS formed during CO release that are important for the bactericidal effect of CORMs of the formulae [Ru(CO)_3_(L)_3_]^2+^.

**Figure 3:**
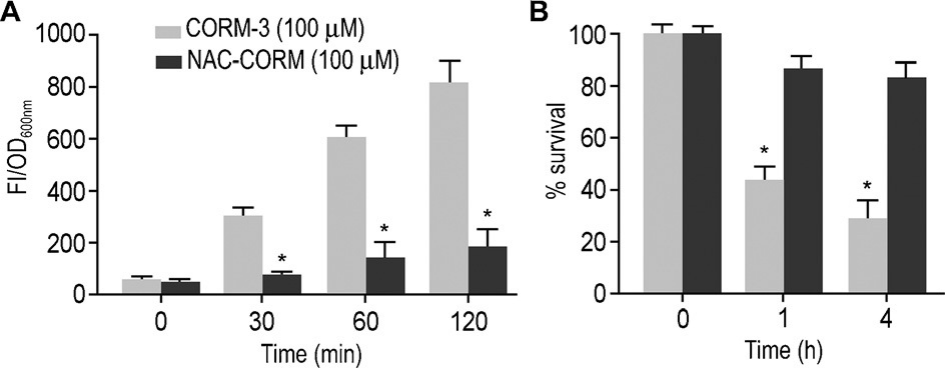
A) Quantification of ROS species in *E. coli* cells treated with 100 μm CORM-3 or NAC-CORM. B) Survival percentage of *E. coli cells* treated with 100 μm of CORM-3 or NAC-CORM. See Supporting Information for full details. Statistically significant differences found after two-way ANOVA are marked as * (*P*<0.05).

CO released from CORM molecules has been extensively demonstrated to possess anti-inflammatory properties.[1] NAC-CORM was designed to not only scavenge the ROS originated during the CO releasing process, but also to enhance the anti-inflammatory properties of CORMs. First, we tested the effect of NAC-CORM and CORM-3 on the production of NO from lipopolysaccharide (LPS)-stimulated RAW264.7 cells. Remarkably, NAC-CORM was able to reduce nitrite levels in the culture by 84 % relative to control cells (Figure 4 A). This shows an enhanced reduction of nitrite levels compared to CORM-3 (58 %) and NAC (60 %) alone. In addition, we also tested the effect of NAC-CORM in the expression levels of tumour necrosis factor (TNF)-α, a key marker of inflammation progression. Both CO and NAC have been reported to influence the expression levels of TNF-α.[2, 23] Treatment of the adenocarcinoma cell line Caco-2 with NAC-CORM showed a synergistic effect promoting a substantial inhibition of the expression of endogenous TNF-α at 4 and 12 h when compared with both CORM-3 and NAC alone at the same concentration (150 μm), as measured by enzyme-linked immunosorbent assay (ELISA) (Figure 4 B). This data provides strong evidence for the synergistic effect of both CO and NAC delivered by the NAC-CORM conjugate here reported.

**Figure 4:**
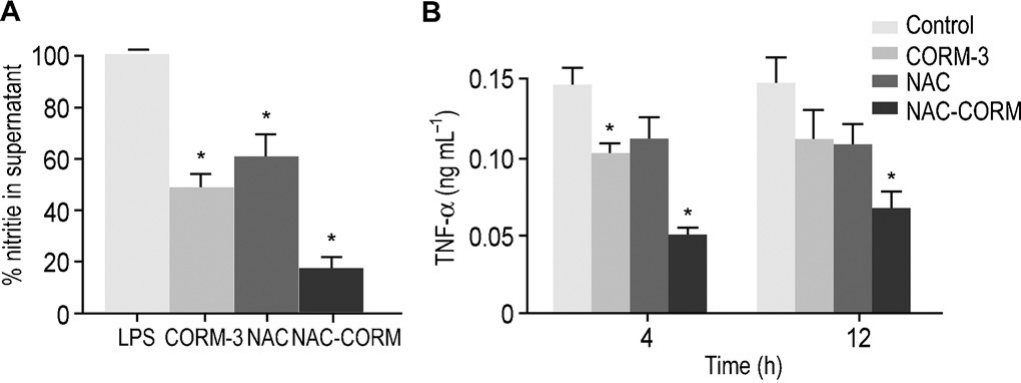
A) Effect of CORM-3, NAC-CORM and NAC at 100 μm on the inhibition of NO production (% control) in LPS-induced RAW264.7 cells. B) Effect of NAC-CORM, CORM-3 and NAC at 150 μm on the expression levels of TNF-α in the supernatant of the adenocarcinoma cell line Caco-2, measured by ELISA. Cytokine expression was measured 4 and 12 h following treatment with 150 μm of NAC-CORM, CORM-3 or NAC. Statistically significant differences found after two-way ANOVA post-hoc test using Bonferroni method are marked as * (*P*<0.05).

In summary, we have produced and characterised a [Ru^II^(CO)_3_Cl_2_(NAC)] complex that simultaneously delivers CO and abolishes ROS formation. Unlike other CORMs of the general formulae [Ru(CO)_3_(L)_3_]^2+^, such as CORM-2 and CORM-3, NAC-CORM favourably reduces the levels of ROS that derive from the hydrolytic instability of such complexes in water. In addition, our studies using the CO-selective probe COP-1 showed evidence that NAC-CORM is more stable compared to CORM-3, as evidenced by a slower CO release kinetics in aqueous solution. Importantly, the NAC and CO delivered after hydrolytic decomposition of the NAC-CORM complex act synergistically showing an enhanced anti-inflammatory activity, as demonstrated by both nitrite reduction and inhibition of expression of TNF-α. Collectively, our data suggests combining of CO releasing motifs based on metal carbonyl scaffolds with ligands that may act synergistically to elicit an enhanced anti-inflammatory response.

## Supporting Information

As a service to our authors and readers, this journal provides supporting information supplied by the authors. Such materials are peer reviewed and may be re-organized for online delivery, but are not copy-edited or typeset. Technical support issues arising from supporting information (other than missing files) should be addressed to the authors.

miscellaneous_information
