# Supplementary material for: An N-Acetyl Cysteine Ruthenium Tricarbonyl Conjugate Enables Simultaneous Release of CO and Ablation of Reactive Oxygen Species
Source: Chemistry. 2015 Aug 28;21(42):14708–12. doi: 10.1002/chem.201502474 (PMC4641457; doi:10.1002/chem.201502474)
Supplement: Supplementary file 1 — miscellaneous_information [file chem0021-14708-sd1.pdf]

CHEMISTRY
A European Journal

Supporting Information

An *N*-Acetyl Cysteine Ruthenium Tricarbonyl Conjugate Enables Simultaneous Release of CO and Ablation of Reactive Oxygen Species

João D. Seixas,[a, b, c] Miguel Chaves-Ferreira,[a] Diana Montes-Grajales,[d, e] Ana M. Gonçalves,[c] Ana R. Marques,[c] Lígia M. Saraiva,[b] Jesus Olivero-Verbel,[e] Carlos C. Romão,[b, c] and Gonçalo J. L. Bernardes*[a, d]

chem_201502474_sm_miscellaneous_information.pdf

Table of Contents

	Page
General considerations	2
Synthetic work	2
Synthesis of $Ru(CO)_3Cl_2(NAC)$	2
Detection of CO or CO_2 using Gas Chromatography	2
Cytotoxicity evaluation	3
Cell culture: Caco-2 and HeLa cells	3
COP-1 fluorescence response to CO measured in buffered aqueous solution	4
COP-1 fluorescence response by confocal microscopy imaging	4
Bacterial growth conditions, ROS quantification and survival	5
LPS stimulation of RAW264.7 cells and nitrite quantification	6
TNF-α modulation by CO release	7
In silico inverse virtual screening	7
Results of *in silico* inverse virtual screening	8
Supporting Table 1	9
IR spectrum of NAC-CORM	15
^{1}H-NMR spectra of NAC-CORM	16
References	21

General Considerations.

Elemental Analysis was performed at the Elemental Analysis Service of the London Metropolitan University by Mr. Stephen Boyer. Infrared spectra were recorded on a Unicam Mattson 7000 FTIR spectrophotometer using KBr pellets. ^{1}H NMR spectra were recorded on a Bruker Avance III 400 mHz. Chemical shifts are quoted in parts per million from $SiMe_4$ (TMS). UV-VIS spectra were acquired in Perkin Elmer Lamda35 spectrophotometer.

Synthetic work.

Reactions were carried out under a nitrogen atmosphere, using common schlenk techniques. Solvents were dried by standard procedures, distilled under N_2 and kept over 4Å molecular sieves, except DMSO that was used as received (*p.a.* from *Panreac*). $[Ru(CO)_3Cl_2]_2$ (CORM-2) and *N*-acetyl-cysteine (NAC) were purchased from *Strem Chemicals* and *Sigma Aldrich*, respectively.

Synthesis of $Ru(CO)_3Cl_2(NAC)$.

N-Acetyl cysteine (0.32 g, 1.53 mmol) was dissolved in MeOH (50 mL) and added to a solution of $[Ru(CO)_3Cl_2]_2$ (CORM-2) (0.39 g, 0.77 mmol) in MeOH (20 mL). The colourless solution was stirred at room temperature for 6 hours. The reaction mixture became slightly turbid, and then was filtered giving a colourless solution that was concentrated *in vacuo*. Diethyl ether was slowly added and precipitation was induced at -30 ºC overnight. The solution was filtered and a white aggregate was obtained. The residue was then washed with diethyl ether (2 x 10 mL) and dried *in vacuo* to give $Ru(CO)_3Cl_2(NAC)$ as a white powder in 53% yield. IR (KBr, cm^{-1}): 2500 (vbr, SH), 2126 (s, C≡O), 2062 (sbr, C≡O); 1749 (s, C=O). Anal. Calcd. for $C_8H_9Cl_2NO_6RuS$: C:22.92; H:2.16; N:3.34; S:7.65, Found: C:23.10; H:2.13; N:3.21; S:7.52. For the detailed ^{1}H NMR characterization of $Ru(CO)_3Cl_2(NAC)$ in D_2O and CD_3OD see Figures 4-8.

Detection of CO or CO_2 using Gas Chromatography.

The CO and CO_2 release assays were performed in 7.5 mL Roth® sample vial equipped with a magnetic stirrer inside and capped with a PTFE rubber or silicone septa and an aluminum cap. PTFE rubber septa were acquired from Sigma Aldrich® and silicone septa from Roth®. The assays were performed in PBS7.4 or H_2O, without light, at room temperature and normal atmospheric air. 250 μL samples were taken

with a Gastight Hamilton® syringe these were injected in a Thermofinnigam Trace GC equipped with a CTR1 column from Alltech™ and a Thermal Conductivity Detector. The column was in a oven at 36 ºC and the GC was operated at a constant pressure mode (111 KPa) with He as a carrier and reference gas with a 30 mL/min flow. The detector was set at constant temperature (150 ºC) and the filament temperature to 250 ºC. The injections were made through a packed column injector (PKD) set at 47 ºC and 111 KPa. CO or CO_2 were quantified using a calibration curve recorded prior to the reaction course. This was done by injecting 25 μL increments of CO up to a final total amount of 2 mL of pure CO gas (Carbon Monoxide 4.7, purity. ≥99.997%; from *Linde Sogas*) to the system and taking samples that were injected in the GC.

Cytotoxicity evaluation.

The toxicity of the compounds was evaluated in a murine macrophage cell line RAW264.7 (ECACC91062702) using the MTT assay. Briefly, RAW264.7 cells were seeded on 96-well plates in DMEM (GIBCO, Invitrogen) medium supplemented with 10% FBS (Fetal Bovine Serum; GIBCO, Invitrogen). Cells were incubated for 24 hours at 37 ºC, 5% CO_2 in a humidified atmosphere before the addition of the compounds to be tested. Compounds were solubilized in water and added to the macrophage cultures at a final concentration of 10, 50 or 100 μM. Cells were incubated for 24 hours in the same conditions described above. The culture medium was replaced by a 1 mg/ml of MTT (3-(4,5-Dimethylthiazol-2-yl)-2,5-diphenyltetrazolium bromide) solution prepared in DMEM medium and the cultures were incubated for 1 hour at 37 ºC, 5% CO_2. The supernatants were discarded and the formazan crystals produced were dissolved in DMSO (Dimethyl sulfoxide). The plates were incubated with gentle shaking for 10 minutes and the absorbance of the medium was read at 550 nm. The absorbance obtained in the control wells was considered as 100% survival.

Cell culture: Caco-2 and HeLa cells.

Caco-2 (ATCC; passage 10-22) and HeLa cells (ECACC; passage 10-22) were routinely grown in a humidified incubator at 37 ºC under 5% CO_2 and split twice a week before reaching confluence using 0.25% trypsin and 1% EDTA. Caco-2 cells were grown as monolayers using MEM GlutaMAX medium (Invitrogen, Life

Technologies), supplemented with 20% heat-inactivated fetal bovine serum (FBS) (Gibco, Life Technologies), 1 mM sodium pyruvate, 200 units/mL penicillin and 200 μg/mL streptomycin (Gibco, Life Technologies). HeLa cells were grown on MEM GlutaMAX medium supplemented with 10% heat-inactivated fetal bovine serum (FBS), 10 mM HEPES (Gibco, Life Technologies), 200 units/mL penicillin and 200 μg/mL streptomycin (Gibco, Life Technologies).

COP-1 fluorescence response to CO measured in buffered aqueous solution.[1]
COP-1 was synthesized according to the literature.[2] Fluorescence of COP-1 in the absence (negative control) or presence of 50 μM NAC-CORM or CORM-3 was determined on different time points using a fluorescence spectrometer, FLS920 (Edinburgh Instruments). A 1 μM solution of COP-1 was prepared in PBS pH 7.4 (without Calcium or Magnesium) from a 5 mM stock solution of COP-1 in DMSO. Experiments were performed at 37 ºC in 500 μL volume. Spectra were taken at 0, 10, 30, 60 and 120 min from 490 to 650 nm following excitation at λ_{ex} = 475 nm.

COP-1 fluorescence response by confocal microscopy imaging.[1]
Images were obtained using a Zeiss LSM 710 confocal Laser Point-Scanning Microscope with a 40X oil objective lens and a numerical aperture of 1.3. COP-1 was excited using an Argon Laser 488 nm and Hoescht 33342 was excited using a Diode Laser 405 nm and were read at green (λ_{em} 500-550 nm) and blue (λ_{em} 420-470 nm), respectively. Cells were imaged at 37 ºC and 5% CO_2 throughout the course of the experiment. $15x10^3$ HeLa cells were seeded in 8-chambered #1.0 Borosilicate coverglass (Lab-Tek), 2 days before the experiment. Culture conditions were the same used for routinely cell passage, using phenol red free MEM medium. Mean of total fluorescence intensity of treated versus untreated cells was compared using representative images of three independent experiments on a per cell basis. Statistical significant differences were analyzed after a two-way ANOVA.

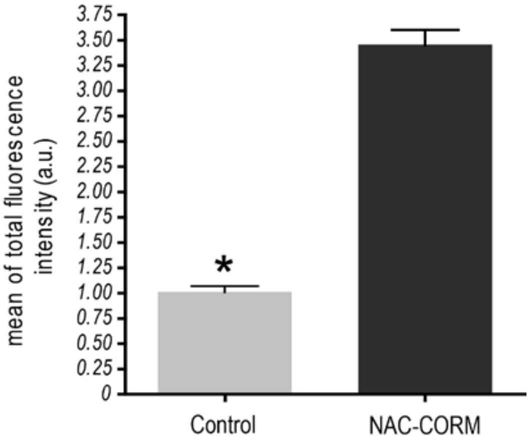

Figure 1. Mean of total fluorescent intensity ± standard error of the mean (SEM) in HeLa cells 30 min after addition of 1 mm COP-1 in the absence (control) or presence of 50 mM mm NAC-CORM. Cells were pre-incubated with NAC-CORM for 30 min prior to COP-1 addition. Mean of total fluorescence intensity is given in arbitrary units (a.u.). Statistically significant differences found after two-way ANOVA are marked as * ($P < 0,05$).

Bacterial growth conditions, ROS quantification and survival

Escherichia coli K-12 ATCC 23716 was grown in minimal medium salts MS (1.3 % (w/v) of Na_2HPO_4, 0.3 % (w/v) of KH_2PO_4, 0.05 % (w/v) of NaCl and 0.1 % (w/v) of NH_4Cl supplemented with 20 mM of glucose, 2 mM of $MgSO_4$, 100 μM of $CaCl_2$ and 0.25 % (w/v) of casamino acids). Overnight cultures of *E. coli* grown in LB was used to inoculate fresh MS medium and incubated at 37 ºC, under the aeration conditions required, until an optical density at 600 nm (OD_{600}) of 0.3.

Endogenous ROS species Cells of *E. coli* were treated for 2 hours, in the presence of 100 μM of NAC-CORM or CORM-3. Cells were harvested, washed twice with phosphate buffer (PBS), and re-suspended in the same buffer. The probe 2',7'-dichlorofluorescein diacetate (Sigma) was then added to the suspended cells, at a final concentration of 10 μM, and fluorescence intensity (FI) was obtained using a Varian Eclipse 96-well spectrofluorimeter (wavelength of excitation at 485 nm and emission at 538 nm). To determine the ROS variation, the FI of cultures with NAC-CORM or CORM-3 were subtracted from those of non-treated cells treated. The FI was normalized in relationship to the A600nm of each culture. These experiments were done in duplicate with at least two different biological samples.

Survival Cells of *E. coli* were exposed to 100 μM of NAC-CORM or CORM-3 and after 4 h the number of viable cells was evaluated by measuring the colony forming

units (cfu/ml) upon plating on agar plates serial dilutions of the various cultures. The percentage of survival was calculated as the number of colonies originated by treated cultures divided by the number of colonies formed upon platting untreated cultures. The experiments result from two independent and error bars representing standard deviation.

LPS stimulation of RAW264.7 cells and nitrite quantification.

Murine macrophages RAW264.7 were seeded into the wells of a 24-well plates in DMEM medium supplemented with 10% FBS. Cells were incubated for 24 hours at 37ºC, 5% CO_2 in a humidified atmosphere. Compounds were added to the cultures at a concentration of 100 μM. At the same time lipopolysaccharide (LPS; L2880, Sigma) was added to the cultures at a concentration of 1 μg/ml. After 24 hours the NO production was determined by quantifying the nitrite in culture supernatants using the Griess reagent. In a 96-well plate, 100 μl of the Griess reagent was mixed with the same volume of culture supernatant and allowed to react for 10 minutes. The absorbance was read at 550 nm. A sodium nitrite standard reference curve was prepared for each assay for accurate quantification of nitrite levels in experimental samples.

TNF-α modulation by CO release.

Growth medium levels of TNF-α was quantified using a TNF-α Mini ELISA Development Kit (Pepro-Tech, sensitivity range of 0.063 to 4 ng/mL) and revealed using TMB substrate reagent set (BD Biosciences) according to the manufacturer's protocol. The absorbance in each well was read at 450 nm by using a microplate reader (Infinite M200 microplate absorbance reader, Tecan). Caco-2 cells were plated at $2.5x10^5$ cell/well in 6 well plates. Cells were incubated with NAC-CORM, CORM-3 or NAC at a concentration of 150 μM, 48 hours after seeding. Supernatants were collected at 4 and 12 post treatment. Statistical significant differences were analyzed after a two-way ANOVA post-hoc test using Bonferroni method. Data are presented in the graphs as mean ± SEM.

In silico **inverse virtual screening.**

The structure of NAC-CORM was drawn in Marvin Sketch Web (http://www.chemaxon.com/marvin/sketch/index.php), using the coordinate bond option for the bonds surrounding the ruthenium atom. It was posteriorly open in Avogadro,[3] in which the hydrogens were added and the nwchem input file generated. The basis used for the optimization procedure in nwchem[4] were DFT B3LYP/6-31G* for all atoms except ruthenium and DFT B3LYP/3-21 g for Ru, the multiplicity was defined as 1, and the number of iterations 200. After the optimization procedure, the Ru^{2+} atom was replaced by Fe^{2+} atom in Avogadro,[3] as Ru is not parametrized in AutoDock Vina, and those atoms have similar periodic properties to be in the same periodic group.[5] The structures of 148 proteins related to tumorogenesis, cell cycle regulation, necrosis and apoptosis were submitted to inverse virtual screening with NAC-CORM. The proteins were previously optimized in Sybyl X-2.0 using the Kollman United and Kollman all atoms force fields by the Powell method, AMBER charges, dielectric constant 1.0, NB cutoff 8.0, maximum interactions 100 and termination gradient 0.001 kcal/mol. The docking grid parameters were established in Autodock Tools,[6] utilizing a grid centered in the macromolecule and covering the whole surface of the protein. For inverse virtual screening, docking calculations were carried out in AutoDock Vina,[7] with exhaustiveness=25, number of modes=20, and by triplicate. The interactions between NAC-CORM and the protein with the best affinity score were predicted using LigPlot version 1.4.3..[8]

Results of *in silico* inverse virtual screening.

To identify the possible targets of NAC-CORM, we performed an inverse virtual screening using AutoDock Vina 1.1.2 that enabled the identification of potential theoretical protein targets of NAC-CORM associated with oxidative stress, tumorigenesis, cell cycle regulation, apoptosis and necrosis. The complete results for the inverse virtual screening are presented in Supporting Table 1. Some proteins exhibited substantial affinity scores for NAC-CORM, in particular, NAD(P)H dehydrogenase quinone 1 (NQO1, -7.8 kcal/mol), a protein related to oxidative stress (Figure 2). This protein is a two-electron reductase involved in chemoprotection and bioactivation of certain antitumor quinones, and in the protection of cellular membranes against oxidative injury.[9-11] Other predicted targets related to oxidative stress included Vitamin D3 receptor (-7.4 kcal/mol), the protein target of the well-known antioxidant and anticarcinogenic vitamin D3,[12] and Catalase (-7.3 kcal/mol), a key enzyme that participates in the destruction of harmful reactive oxygen species, converting hydrogen peroxide into water.[13] Other proteins related to apoptosis, cell cycle regulation and tumorigenesis also exhibited good affinity scores for NAC-CORM (≤-7.0 kcal/mol), such as Adiponectin (-7.8 kcal/mol), Nuclear receptor ROR-gamma (-7.5 kcal/mol), Apoptotic protease-activating factor 1 (-7.4 kcal/mol), FADD (-7.4 kcal/mol) and nucleoside diphosphate kinase A (-7.4 kcal/mol). In performing this virtual screen we were able to identify a number of the potential targets of NAC-CORM such as those involved in oxidative stress conditions.

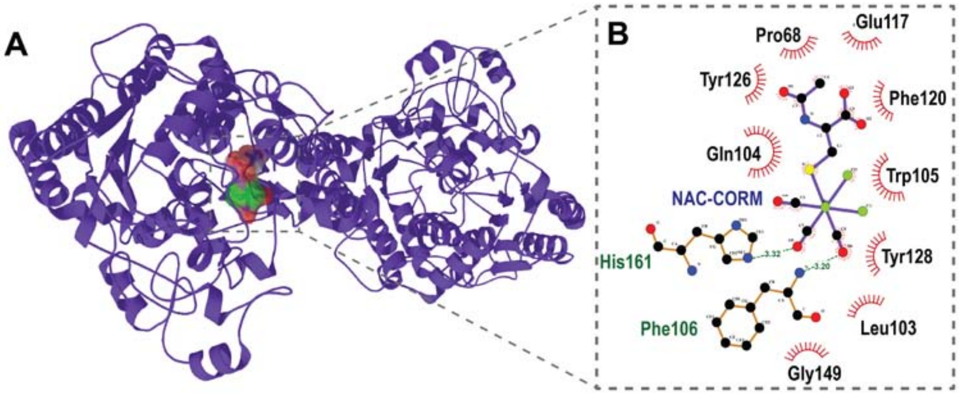

Figure 2. 3D-View of the **A:** NAC-CORM/NQO1 (PDB: 1D4A) complex and **B:** contact residues.

Supporting Table 1: Affinity scores obtained for the docking between NAC-CORM and proteins involved in different signaling pathways.

Short name	Name	Uniprot	PDB	Run1	Run2	Run3	Average	Standard Deviation
NQO1	NAD(P)H dehydrogenase [quinone] 1	P15559	1D4A	-7.6	-7.7	-8	-7.8	0.2
ADIPOQ	Adiponectin	Q15848	4DOU	-7.8	-7.7	-7.8	-7.8	0.1
RORC	Nuclear receptor ROR-gamma	P51449	3L0L	-7.5	-7.4	-7.5	-7.5	0.1
APAF1	Apoptotic protease-activating factor 1	O14727	1Z6T	-7.9	-7.3	-7.1	-7.4	0.4
FADD	Protein FADD	Q13158	2GF5	-7.3	-7.3	-7.6	-7.4	0.2
VDR	Vitamin D3 receptor	P11473	1IE9	-7.3	-7.4	-7.4	-7.4	0.1
NME1	Nucleoside diphosphate kinase A	P15531	1UCN	-7.2	-7.5	-7.4	-7.4	0.2
CAT	Catalase	P04040	1DGF	-6.8	-6.5	-8.6	-7.3	1.1
ODC1	Ornithine decarboxylase	P11926	2OO0	-7.3	-7.3	-7.2	-7.3	0.1
MMP9	Matrix metalloproteinase-9	P14780	4H3X	-7.4	-7.2	-7.1	-7.2	0.2
TIMP1	Metalloproteinase inhibitor 1	P01033	3V96	-7.1	-7.3	-7.3	-7.2	0.1
RB1	Retinoblastoma-associated protein	P06400	4ELJ	-7.3	-7.1	-7.3	-7.2	0.1
FGFR2	Fibroblast growth factor receptor 2	P21802	3B2T	-7.1	-7.2	-7.2	-7.2	0.1
IRS1	Insulin receptor substrate 1	P35568	1QQG	-7.3	-7.1	-7.1	-7.2	0.1
CXCR4	C-X-C chemokine receptor type 4	P61073	3ODU	-7.1	-7.1	-7.3	-7.2	0.1
PIK3CA	Phosphatidylinositol 4,5-bisphosphate 3-kinase catalytic subunit alpha isoform	P42336	3HHM	-6.9	-7.3	-7.2	-7.1	0.2
RXRB	Retinoic acid receptor RXR-beta	P28702	1H9U	-7.2	-6.9	-7.2	-7.1	0.2
PIK3CG	Phosphatidylinositol 4,5-bisphosphate 3-kinase catalytic subunit gamma isoform	P48736	3MJW	-7	-7.1	-7.2	-7.1	0.1
CYP1B1	Cytochrome P450 1B1	Q16678	3PM0	-7.2	-6.8	-7.3	-7.1	0.3
RORA	Nuclear receptor ROR-alpha	P35398	1N83	-7.1	-7.1	-7.1	-7.1	0.0
ABL1	Tyrosine-protein kinase ABL1	P00519	2FO0	-6.8	-7.2	-7.2	-7.1	0.2
SRC	Proto-oncogene tyrosine-protein kinase Src	P12931	2H8H	-7.1	-7.1	-7	-7.1	0.1
GSTM1	Glutathione S-transferase Mu 1	P09488	1GTU	-7.2	-6.9	-7	-7.0	0.2
GSR	Glutathione reductase, mitochondrial	P00390	1GRB	-7.3	-6.4	-7.3	-7.0	0.5
RARB	Retinoic acid receptor beta	P10826	4DM6	-6.8	-7.3	-6.7	-6.9	0.3
LCK	Tyrosine-protein kinase Lck	P06239	1X27	-6.8	-7	-6.9	-6.9	0.1
MTOR	Serine/threonine-protein kinase Mtor	P42345	4JSN	-7	-6.8	-6.9	-6.9	0.1

KDR	Vascular endothelial growth factor receptor 2	P35968	3VO3	-7	-7.1	-6.6	-6.9	0.3
APOD	Apolipoprotein D	P05090	2HZQ	-6.9	-6.8	-6.8	-6.8	0.1
KIT	Mast/stem cell growth factor receptor Kit	P10721	1T45	-6.8	-6.9	-6.8	-6.8	0.1
NOS2	Nitric oxide synthase, inducible	P35228	3E7G	-6.9	-6.9	-6.7	-6.8	0.1
PPARD	Peroxisome proliferator-activated receptor delta	Q03181	2AWH	-6.8	-6.8	-6.9	-6.8	0.1
RARA	Retinoic acid receptor alpha	P10276	3KMR	-6.8	-6.8	-6.8	-6.8	0.0
MSH2	DNA mismatch repair protein Msh2	P43246	2O8B	-7	-6.9	-6.5	-6.8	0.3
RXRG	Retinoic acid receptor RXR-gamma	P48443	2GL8	-6.7	-6.7	-6.9	-6.8	0.1
HTRA2	Serine protease HTRA2, mitochondrial	O43464	1LCY	-6.8	-6.7	-6.7	-6.7	0.1
MLH1	DNA mismatch repair protein Mlh1	P40692	3RBN	-6.6	-6.8	-6.8	-6.7	0.1
NTRK1	High affinity nerve growth factor receptor	P04629	4GT5	-6.7	-6.7	-6.7	-6.7	0.0
RPS6KA1	Ribosomal protein S6 kinase alpha-1	Q15418	2WNT	-7	-7.1	-6	-6.7	0.6
MAPK14	Mitogen-activated protein kinase 14	Q16539	2Y8O	-6.7	-6.7	-6.7	-6.7	0.0
NR5A2	Nuclear receptor subfamily 5 group A member 2	O00482	1YUC	-6.3	-7.1	-6.6	-6.7	0.4
STRADA/MO25	STRAD/MO25 Complex	Q7RTN6/Q9Y37 6	3GNI	-6.6	-6.8	-6.6	-6.7	0.1
F2	Prothrombin	P00734	1A3B	-6.6	-6.7	-6.6	-6.6	0.1
SERPINB5	Serpin B5	P36952	1WZ9	-6.6	-6.7	-6.6	-6.6	0.1
PALB2	Partner and localizer of BRCA2	Q86YC2	2W18	-6.2	-7.1	-6.5	-6.6	0.5
GSTP1	Glutathione S-transferase P	P09211	3N9J	-6.6	-6.6	-6.6	-6.6	0.0
FLT1	Vascular endothelial growth factor receptor 1	P17948	3HNG	-6.6	-6.6	-6.6	-6.6	0.0
KCIP-1	14-3-3 protein zeta/delta	P63104	1QJB	-6.7	-6.5	-6.6	-6.6	0.1
PPP2CA	Serine/threonine-protein phosphatase 2A catalytic subunit alpha isoform	P67775	3FGA (chain c)	-6.6	-6.6	-6.6	-6.6	0.0
SERPINE1	Plasminogen activator inhibitor 1	P05121	1A7C	-6.3	-7	-6.3	-6.5	0.4
RPS6KB1	Ribosomal protein S6 kinase beta-1	P23443	4L3J	-7.3	-5.8	-6.5	-6.5	0.8
Fas/FADD	Fas-FADD death domain complex	Q13158, P25445	3EZQ	-6.8	-6.7	-6.1	-6.5	0.4
ERBB2	Receptor tyrosine-protein kinase erbB-2	P04626	1N8Z	-6.3	-6.7	-6.5	-6.5	0.2
RARG	Retinoic acid receptor gamma	P13631	2LBD	-6.5	-6.6	-6.4	-6.5	0.1
HMOX1	human heme oxygenase-1	P09601	3CZY	-6.5	-6.6	-6.4	-6.5	0.1

NR1D1	Nuclear receptor subfamily 1 group D member 1	P20393	1A6Y	-6.4	-6.5	-6.5	-6.5	0.1
AKT1	RAC-alpha serine/threonine-protein kinase	P31749	4GV1	-6.7	-6.3	-6.4	-6.5	0.2
TF	Serotransferrin	P02787	1A8E	-6.5	-6.4	-6.4	-6.4	0.1
MAPK3	Mitogen-activated protein kinase 3	P27361	2ZOQ	-6.2	-6.7	-6.4	-6.4	0.3
EPHB2	Ephrin type-B receptor 2	P29323	2QBX	-6.5	-6.6	-6.2	-6.4	0.2
CASP3	Caspase-3	P42574	3H0E	-6.4	-6.5	-6.4	-6.4	0.1
MCL1	Induced myeloid leukemia cell differentiation protein Mcl-1	Q07820	2KBW	-6.4	-6.5	-6.4	-6.4	0.1
CHKA	Choline kinase alpha	P35790	2CKO	-6.4	-6.4	-6.4	-6.4	0.0
VHL	Von Hippel-Lindau disease tumor suppressor	P40337	1LM8	-6.3	-6.5	-6.4	-6.4	0.1
GSTK1	Glutathione S-transferase kappa 1	Q9Y2Q3	3RPP	-6.4	-6.4	-6.4	-6.4	0.0
PLAU	Urokinase-type plasminogen activator	P00749	1C5Y	-6.7	-6.2	-6.3	-6.4	0.3
MMP2	72 kDa type IV collagenase	P08253	1CK7	-6.6	-6.3	-6.3	-6.4	0.2
HNF4A/SRC1	HNF4a LBD in complex with the coactivator SRC-1 peptide	P41235, Q15788	1PZL	-6.3	-6.4	-6.5	-6.4	0.1
CASP9	Caspase-9	P55211	1NW9	-6.3	-6.6	-6.3	-6.4	0.2
14-3-3 protein theta	14-3-3 protein T-cell	P27348	2BTP	-6.3	-6.4	-6.4	-6.4	0.1
HBA1	Hemoglobin subunit alpha	P69905	1A01	-6.2	-6.4	-6.4	-6.3	0.1
ARF-BP1	E3 ubiquitin-protein ligase HUWE1	Q7Z6Z7	3H1D	-6.4	-6.1	-6.5	-6.3	0.2
RELA	Transcription factor p65	Q04206	2O61	-6.4	-6	-6.5	-6.3	0.3
CIB1	Calcium and integrin-binding protein 1	Q99828	2L4H	-6.3	-6.3	-6.3	-6.3	0.0
CCNB1	G2/mitotic-specific cyclin-B1	P14635	2B9R	-6.3	-6.2	-6.3	-6.3	0.1
PPARG	Peroxisome proliferator-activated receptor gamma	P37231	3LMP	-6.3	-6.3	-6.2	-6.3	0.1
CASP8	Caspase-8	Q14790	2C2Z	-6	-6.4	-6.4	-6.3	0.2
PPARA	Peroxisome proliferator-activated receptor alpha	Q07869	2P54	-5.9	-7.3	-5.6	-6.3	0.9
CD8A	T-cell surface glycoprotein CD8 alpha chain	P01732	1AKJ	-6.3	-6.2	-6.2	-6.2	0.1
ITK	Tyrosine-protein kinase ITK/TSK	Q08881	3T9T	-6.4	-6.1	-6.2	-6.2	0.2
UBE2S	Ubiquitin-conjugating enzyme E2 S	Q16763	1ZDN	-6.3	-6.2	-6.2	-6.2	0.1
PRPF31	U4/U6 small nuclear ribonucleoprotein Prp31	Q8WWY3	2OZB	-6.2	-6	-6.5	-6.2	0.3
IL1B	Interleukin-1 beta	P01584	1L2H	-6	-6.3	-6.3	-6.2	0.2

ESR1	Estrogen receptor	P03372	3ERT	-6.2	-6.2	-6.2	-6.2	0.0
F2R	Proteinase-activated receptor 1	P25116	3VW7	-6.1	-6.4	-6.1	-6.2	0.2
CTNNB1	Catenin beta-1	P35222	1G3J	-6	-6.3	-6.3	-6.2	0.2
AIF1	Allograft inflammatory factor 1	P55008	2G2B	-6.2	-6.2	-6.2	-6.2	0.0
PPP2R5C	Serine/threonine-protein phosphatase 2A 56 kDa regulatory subunit gamma isoform	Q13362	3FGA	-6.2	-6.3	-6.1	-6.2	0.1
BAD	Bcl2-associated agonist of cell death	Q92934	1G5J	-6.4	-6.1	-6.1	-6.2	0.2
DCTN6	Dynactin subunit 6	O00399	3TV0	-6.1	-6.2	-6.2	-6.2	0.1
SOD2	Superoxide dismutase [Mn], mitochondrial	P04179	1AP6	-6.2	-6.2	-6.1	-6.2	0.1
PTEN	Phosphatidylinositol 3,4,5-trisphosphate 3-phosphatase and dual-specificity protein phosphatase PTEN	P60484	1D5R	-6.1	-6.1	-6.3	-6.2	0.1
TNF	Tumor necrosis factor	P01375	1A8M	-6	-6.3	-6	-6.1	0.2
IFN-gamma	INTERFERON-GAMMA	P01579	1FYH	-6.4	-6	-5.9	-6.1	0.3
ANXA5	Annexin A5	P08758	1ANX	-6.3	-6.1	-5.9	-6.1	0.2
PRL	Prolactin	P01236	1RW5	-6	-6	-6.2	-6.1	0.1
CDKN1A	Cyclin-dependent kinase inhibitor 1	P38936	2ZVV	-6	-6.1	-6	-6.0	0.1
IGF1R	Insulin-like growth factor 1 receptor	P08069	1IGR	-6	-6	-6	-6.0	0.0
IL10	Interleukin-10	P22301	2ILK	-5.9	-6	-6.1	-6.0	0.1
14-3-3E	14-3-3 protein epsilon	P62258	2BR9	-6	-6	-6	-6.0	0.0
CTSB	Cathepsin B	P07858	3CBJ	-6	-6	-5.9	-6.0	0.1
MET	Hepatocyte growth factor receptor	P08581	1R0P	-5.9	-6	-6	-6.0	0.1
BCL2	Apoptosis regulator Bcl-2	P10415	1G5M	-6	-6	-5.9	-6.0	0.1
MMP14	Matrix metalloproteinase-14	P50281	3C7X	-5.9	-6.1	-5.9	-6.0	0.1
NR3C1	Glucocorticoid receptor	P04150	3E7C	-5.9	-6	-5.9	-5.9	0.1
BAX	Apoptosis regulator BAX	Q07812	1F16	-6	-5.9	-5.9	-5.9	0.1
MCL1/B2L11	Mcl-1 in complex with Bim BH3 mutant I2dY	Q07820, O43521	3KJ0	-5.9	-5.9	-6	-5.9	0.1
LEP	Leptin	P41159	1AX8	-5.7	-6	-6.1	-5.9	0.2
KRAS	GTPase KRas	P01116	4EPW	-5.9	-5.9	-5.9	-5.9	0.0
HRAS	GTPase HRas	P01112	1AA9	-5.7	-6	-6	-5.9	0.2
CHEK2	Serine/threonine-protein kinase Chk2	O96017	2W0J	-5.8	-5.9	-5.9	-5.9	0.1

CDK2	Cyclin-dependent kinase 2	P24941	1AQ1	-6	-5.8	-5.8	-5.9	0.1
IL4	Interleukin-4	P05112	2B8U	-5.9	-5.8	-5.8	-5.8	0.1
ROS1	Proto-oncogene tyrosine-protein kinase ROS	P08922	3ZBF	-6	-5.7	-5.8	-5.8	0.2
MAPK1	Mitogen-activated protein kinase 1	P28482	3I60	-5.9	-5.8	-5.8	-5.8	0.1
TRIM24	Transcription intermediary factor 1-alpha	O15164	3O36	-5.7	-5.8	-5.9	-5.8	0.1
CD44	CD44 antigen	P16070	1POZ	-5.7	-5.9	-5.7	-5.8	0.1
CCNA2	Cyclin-A2	P20248	2CCH	-5.8	-5.8	-5.7	-5.8	0.1
ESR2	Estrogen receptor beta	Q92731	1QKM	-5.9	-5.8	-5.6	-5.8	0.2
MAPK8	Mitogen-activated protein kinase 8	P45983	2XRW	-5.9	-5.6	-5.6	-5.7	0.2
IL1RN	Interleukin-1 receptor antagonist protein	P18510	1IRP	-5.6	-5.6	-5.8	-5.7	0.1
PLG	Plasminogen	P00747	1KI0	-5.4	-5.7	-5.7	-5.6	0.2
NFKBIA	NF-kappa-B inhibitor alpha	P25963	1IKN	-5.4	-5.7	-5.7	-5.6	0.2
TNFSF11	Tumor necrosis factor ligand superfamily member 11	O14788	3URF	-5.6	-5.6	-5.6	-5.6	0.0
FN1	Fibronectin	P02751	3T1W	-5.6	-5.8	-5.4	-5.6	0.2
MDM2	E3 ubiquitin-protein ligase Mdm2	Q00987	2AXI	-5.6	-5.6	-5.6	-5.6	0.0
GH1	Somatotropin	P01241	1AXI	-5.6	-5.5	-5.6	-5.6	0.1
CDKN2A	Cyclin-dependent kinase inhibitor 2A, isoforms 1/2/3	P42771	1A5E	-5.6	-5.4	-5.7	-5.6	0.2
DLC8	PROTEIN INHIBITOR OF NEURONAL NITRIC OXIDE SYNTHASE	P63167	1CMI	-5.6	-5.4	-5.6	-5.5	0.1
ERBB2IP	Erb-B2 INTERACTING PROTEIN	Q96RT1	1MFG	-5.6	-5.5	-5.5	-5.5	0.1
CD4	T-cell surface glycoprotein CD4	P01730	1CDY	-5.5	-5.5	-5.5	-5.5	0.0
FGF2	Fibroblast growth factor 2	P09038	1BAS	-5.5	-5.5	-5.5	-5.5	0.0
LGALS1	Galectin-1	P09382	3OY8	-5.5	-5.5	-5.5	-5.5	0.0
BIRC5	Baculoviral IAP repeat-containing protein 5	O15392	2QFA	-5.5	-5.2	-5.7	-5.5	0.3
BCLX	Bcl-2-like protein 1	Q07817	1R2D	-5.4	-5.5	-5.5	-5.5	0.1
RAC1	Ras-related C3 botulinum toxin substrate 1	P63000	1MH1	-5.5	-5.4	-5.4	-5.4	0.1
PARK7	Protein DJ-1	Q99497	1Q2U	-5.5	-5.5	-5.3	-5.4	0.1
CRADD	Death domain-containing protein CRADD	P78560	2O71	-5.2	-5.5	-5.6	-5.4	0.2
SMAC	Diablo homolog, mitochondrial	Q9NR28	1FEW	-5.6	-5.3	-5.4	-5.4	0.2
PCNA	Proliferating cell nuclear antigen	P12004	1U7B	-5.6	-5.2	-5.3	-5.4	0.2

IL2	Interleukin-2	P60568	1M47	-5.2	-5.2	-5.6	-5.3	0.2
NBR1	Next to BRCA1 gene 1 protein	Q14596	2BKF	-5.3	-5.3	-5.3	-5.3	0.0
HNF4A	Hepatocyte nuclear factor 4-alpha	P41235	1PZL	-5.1	-5.3	-5.2	-5.2	0.1
RAD51	DNA repair protein RAD51 homolog 1	Q06609	1N0W	-5.1	-5.2	-5.2	-5.2	0.1
IL6	Interleukin-6	P05231	1ALU	-5.1	-5.2	-5.1	-5.1	0.1
ICAM1	Intercellular adhesion molecule 1	P05362	1IAM	-5.1	-5	-5.1	-5.1	0.1
NOXA	Phorbol-12-myristate-13-acetate-induced protein 1	Q13794	3MQP	-4.8	-4.1	-4.8	-4.6	0.4
BCL2A1	Bcl-2-related protein A1	Q16548	2VM6	-4.3	-4.1	-4.1	-4.2	0.1

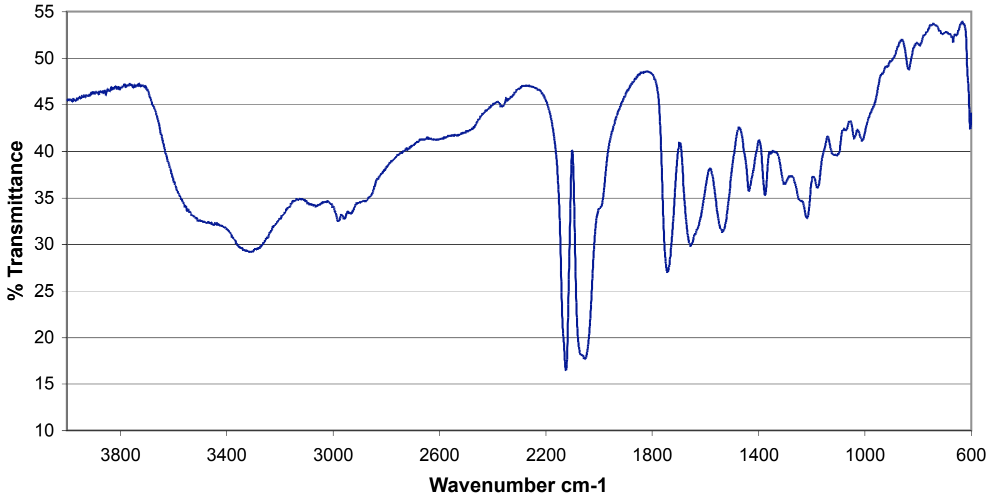

Figure 3. IR spectrum of $RuCl_2(CO)_3$(NAC) in KBr pellet.

^{1}H-NMR spectrum of $RuCl_2(CO)_3(NAC)$ in CD_3OD

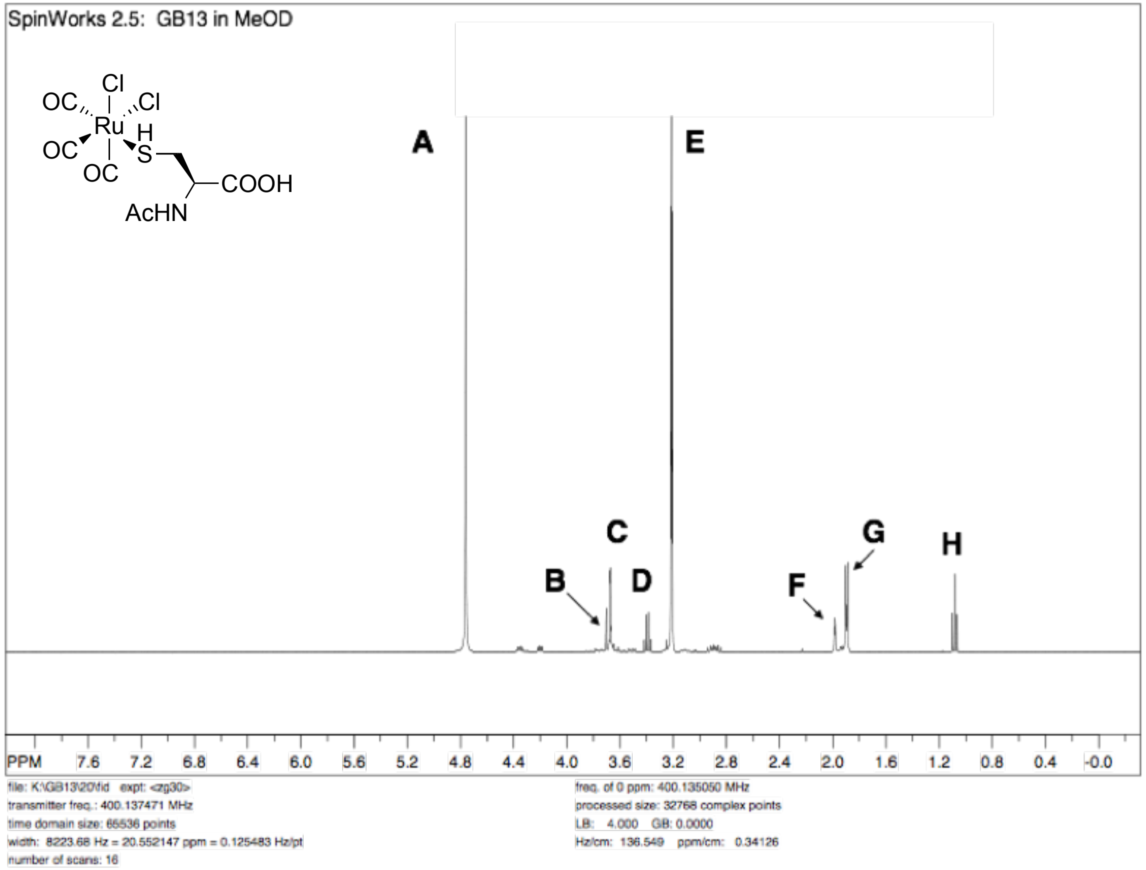

Figure 4. ^{1}H NMR spectrum of $RuCl_2(CO)_3(NAC)$ in CD_3OD. Peak A assigned to H_2O in CD_3OD; peaks D and H assigned to residual Et_2O (δ ppm 3.49 and 1.18); peak E is assigned to CD_3 from CD_3OD (δ ppm 3.31). Peaks F and G assigned to CH_3 groups in two isomers. Peaks B and C assigned to CH_2 groups of 2 isomers. Compare with spectra taken in D_2O (below) and spectrum of NAC in CD_3OD (below)

^{1}H-NMR spectrum of $RuCl_2(CO)_3(NAC)$ in D_2O

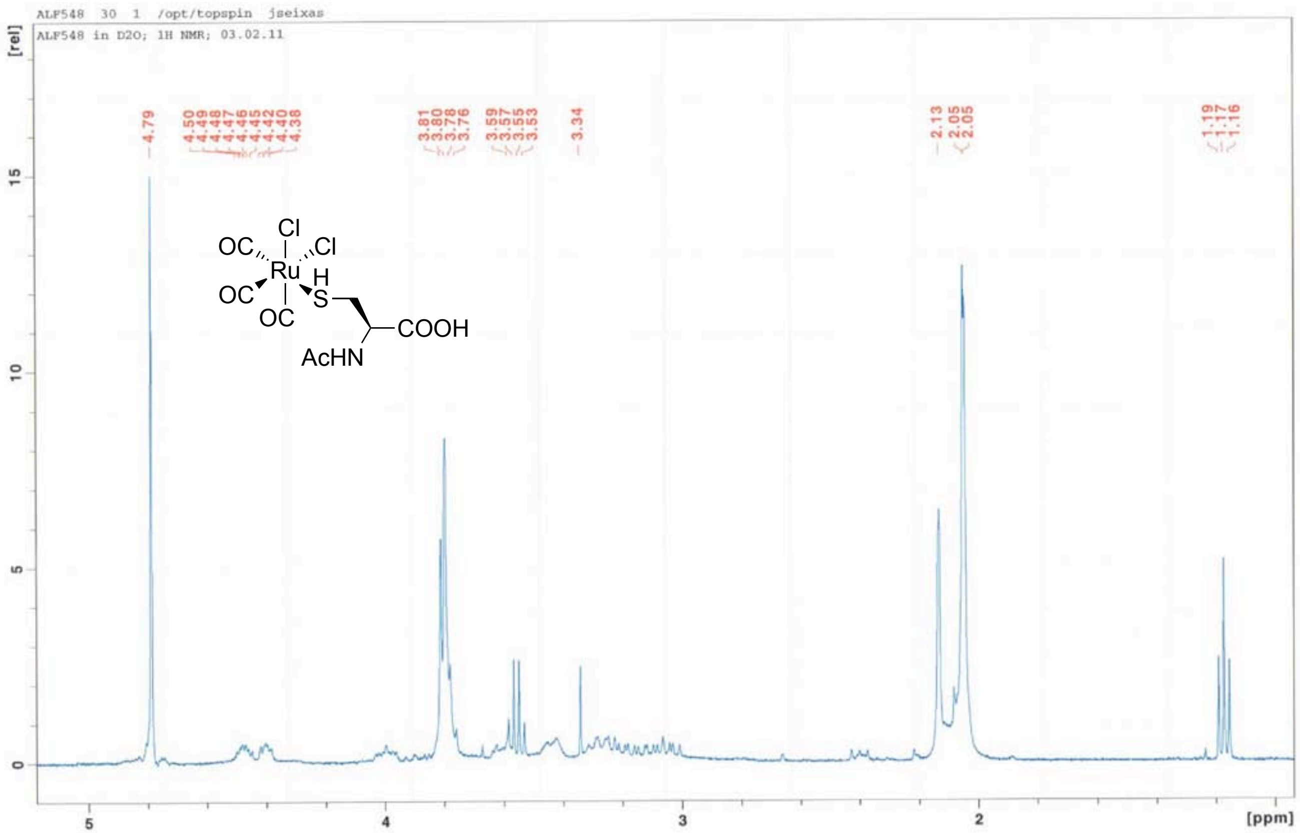

Figure 5. ^{1}H-NMR spectrum of $RuCl_2(CO)_3(NAC)$ in D_2O. Assignments; Et_2O (δ ppm 1.17, 3.56); CH_3OH (δ ppm 3.34; residual in NAC-CORM) and H_2O (δ ppm 4.79); two acetyl CH_3 signals (δ ppm 2.05, 2.13); two CH_2 signals (δ ppm 3.76-3.81); one CH signals (δ ppm 4.40-4.47). See Figure 4 for integration.

^{1}H-NMR spectrum of $RuCl_2(CO)_3(NAC)$ in D_2O (with integrals)

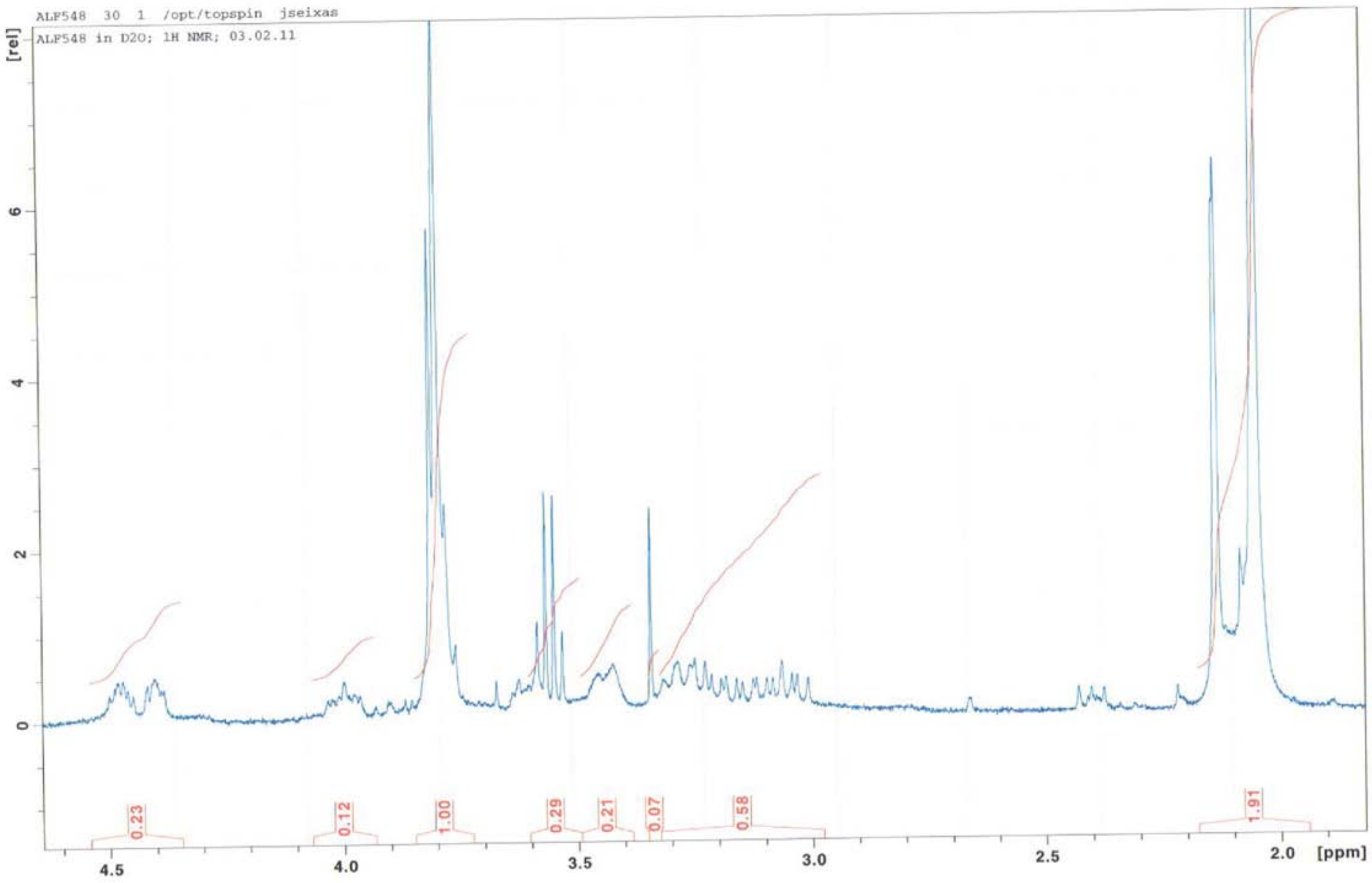

Figure 6. ^{1}H-NMR spectrum of $RuCl_2(CO)_3(NAC)$ in D_2O; integration of spectrum in Figure 3.

^{1}H-NMR spectrum of NAC in D_2O

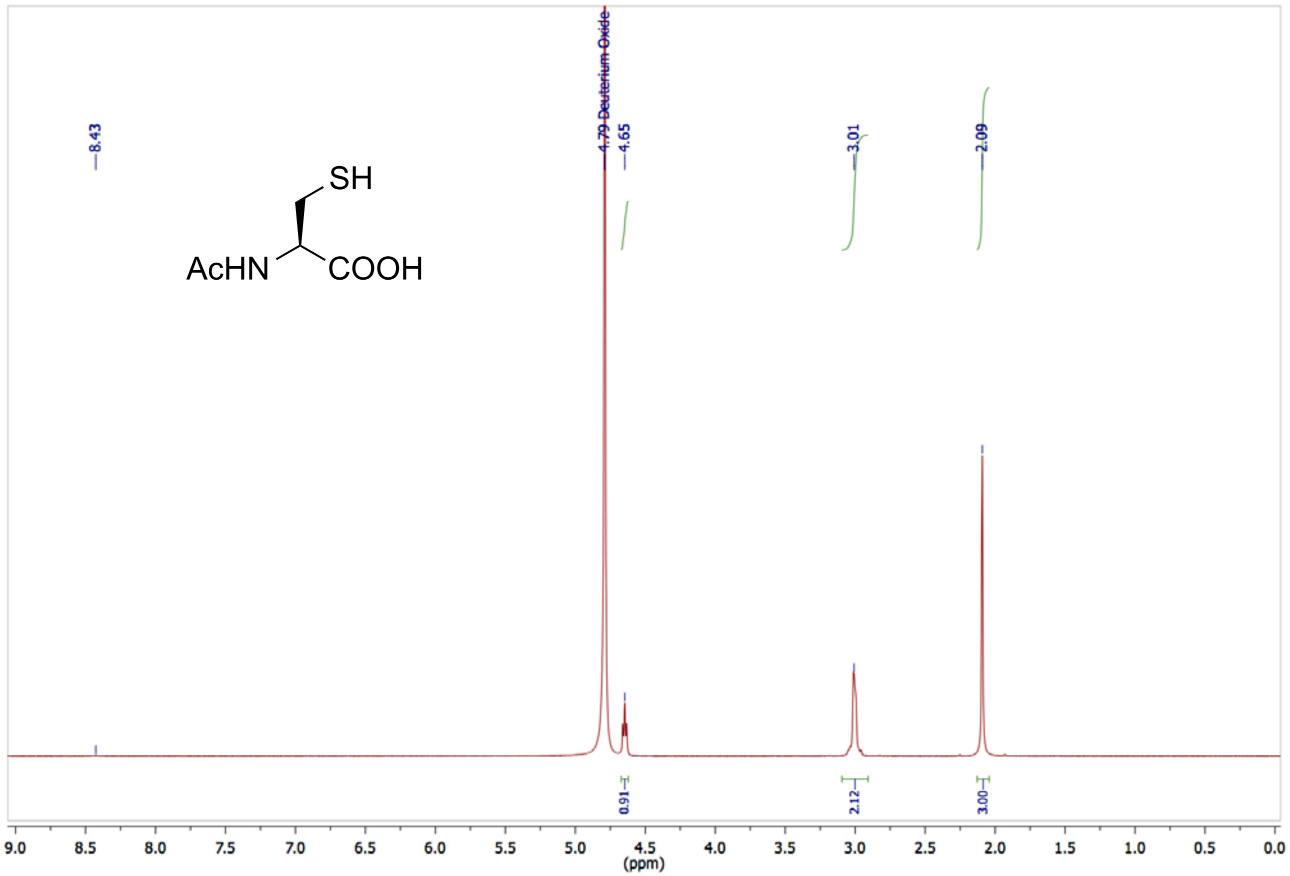

Figure 7. ^{1}H-NMR spectrum of NAC in D_2O.

^{1}H-NMR spectrum of NAC in CD_3OD

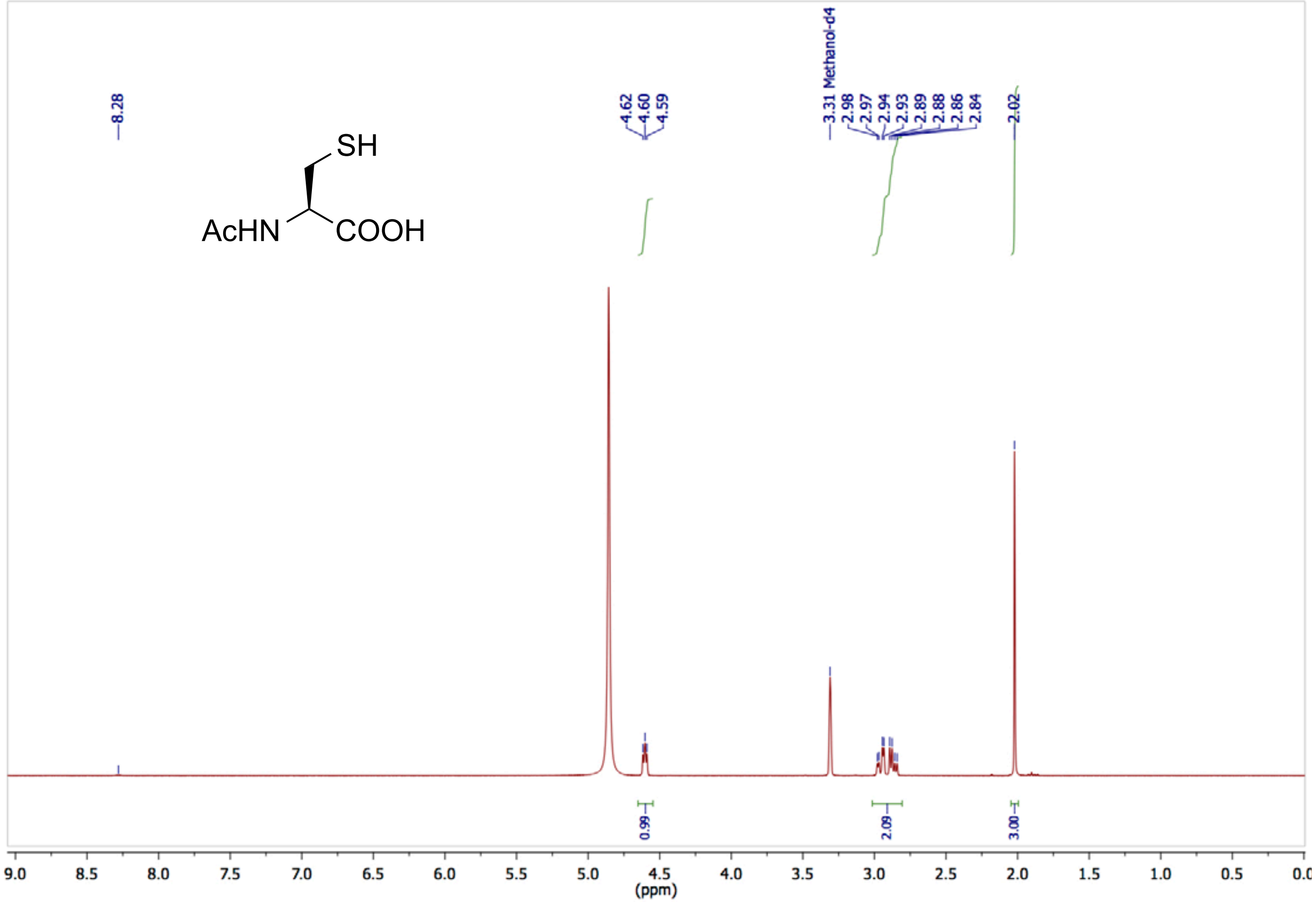

Figure 8. ^{1}H-NMR spectrum of NAC in CD_3OD.

References

[1] M. Chaves-Ferreira, I. S. Albuquerque, D. Matak-Vinkovic, A. C. Coelho, S. M. Carvalho, L. M. Saraiva, C. C. Romão, G. J. L. Bernardes, *Angew. Chem. Int. Ed.* **2015**, *54*, 1172-1175.

[2] B. W. Michel, A. R. Lippert, C. J. Chang, *J. Am. Chem. Soc.* **2012**, *134*, 15668-15671.

[3] M. Hanwell, D. Curtis, D. Lonie, T. Vandermeersch, E. Zurek, G. Hutchison, *J. Cheminfor.* **2012**, *4*, 17-.

[4] M. Valiev, E. J. Bylaska, N. Govind, K. Kowalski, T. P. Straatsma, H. J. J. Van Dam, D. Wang, J. Nieplocha, E. Apra, T. L. Windus, W. A. de Jong, *Comput. Phys. Commun.* **2010**, *181*, 1477-1489.

[5] N. P. Cook, M. Ozbil, C. Katsampes, R. Prabhakar, A. A. Martí, *J. Am. Chem. Soc.* **2013**, *135*, 10810-10816.

[6] G. M. Morris, R. Huey, W. Lindstrom, M. F. Sanner, R. K. Belew, D. S. Goodsell, A. J. Olson, *J. Comput. Chem.* **2009**, *30*, 2785-2791.

[7] O. Trott, A. J. Olson, *J. Comput. Chem.* **2010**, *31*, 455-461.

[8] A. C. Wallace, R. A. Laskowski, J. M. Thornton, *Protein Eng.* **1995**, *8*, 127-134.

[9] A. T. Dinkova-Kostova, P. Talalay, *Arch. Biochem. Biophys.* **2010**, *501*, 116-123.

[10] D. Ross, J. K. Kepa, S. L. Winski, H. D. Beall, A. Anwar, D. Siegel, *Chem. Biol. Interact.* **2000**, *129*, 77-97.

[11] G. Wang, R. J. Maier, *Infect. Immun.* **2004**, *72*, 1391-1396.

[12] R. Karmakar, S. Banik, M. Chatterjee, *J. Exp. Ther. Oncol.* **2002**, *2*, 193-199.

[13] T. P. Ribeiro, C. Fernandes, K. V. Melo, S. S. Ferreira, J. A. Lessa, R. W. A. Franco, G. Schenk, M. D. Pereira, A. Horn Jr, *Free Radic. Biol. Med.* **2015**, *80*, 67-76.
